# A comparison of COVID‐19 vaccination status among pregnant Israeli Jewish and Arab women and psychological distress among the Arab women

**DOI:** 10.1111/nhs.12929

**Published:** 2022-02-24

**Authors:** Orit Taubman – Ben‐Ari, Efrat Weiss, Salam Abu‐Sharkia, Enas Khalaf

**Affiliations:** ^1^ The Louis and Gabi Weisfeld School of Social Work Bar‐Ilan University Ramat Gan Israel; ^2^ Me and my Baby Virtual Community Israel

**Keywords:** anxiety, COVID‐19, pregnancy, psychological distress, multicultural health, vaccination

## Abstract

The public debate surrounding the COVID‐19 vaccine is especially intense regarding pregnant women, who are concerned with its effects on themselves and their fetus, and a vulnerable at‐risk population for psychological distress. We aimed at describing differences in vaccination status between pregnant Jewish and Arab women and understanding factors contributing to psychological distress among Arab women. Pregnant women (*n* = 860) aged 19–46 completed self‐report questionnaires during the national vaccination program (March–April 2021). The questionnaires related to background, COVID‐19‐related vaccination status and intentions in this regard, COVID‐19‐related anxiety, and the Mental Health Inventory—Short Form. Data were analyzed using descriptive statistics, *t*‐ and chi‐square tests, Pearson correlations, and a hierarchical regression. Considerably fewer Jewish women had been infected and more were vaccinated than Arab women. Poorer health, lower economic status, being a mother, not being vaccinated, higher anxiety over economic damage, a family member being infected, delivery, and raising the baby contributed to higher distress. Findings offer novel insights for nurses in their efforts to encourage vaccination, highlighting the need to understand women's concerns during the vulnerable period of pregnancy.

**Key points**

- More Jewish women had recovered from COVID‐19 and more were vaccinated than Arab women.
- Not being vaccinated contributed to higher psychological distress among Arab pregnant women.
- Higher anxiety over economic damage, a family member being infected, delivery, and raising the baby contributed to higher distress.

## 1 INTRODUCTION

The global despair and frustration brought about by the worldwide outbreak of COVID‐19 has been replaced by cautious optimism with the discovery of several effective vaccines. In December 2020, approximately one year after the start of the pandemic, Israel launched a national vaccination project, aiming to inoculate the entire adult population over the age of 16 with the Pfizer vaccine as quickly as possible, beginning with the oldest citizens. However, questions were raised as to whether or not pregnant women should receive the vaccine given the limited knowledge available concerning its effectiveness and the risks for the mother and fetus. These were accompanied by evidence of the harmful effects of COVID‐19 among pregnant women (e.g., Huntley et al., 2020; World Association of Perinatal Medicine [WAPM], 2021), which received considerable exposure in the national media.

## 2 BACKGROUND

Large‐scale cohort studies found that infection during pregnancy was associated with a 0.8% rate of maternal mortality, 11.1% rate of admission to intensive care units, and increased rates of complications leading to a higher proportion of both cesarean sections and preterm deliveries (Huntley et al., 2020; WAPM, 2021). Moreover, although studies showed that vertical transmission from mother to fetus seemed to be negligible (Huntley et al., 2020; WAPM, 2021), they also indicated that the earlier in pregnancy the infection occurred, the greater the risk of adverse fetal outcomes (Di Mascio et al., 2020).

In view of this information, pregnant women were considered an at‐risk population, and most experts worldwide favored including them in the vaccination programs (Dashraath et al., 2020; Whitehead & Walker, 2020), a recommendation endorsed by the International Federation of Gynecology and Obstetrics (FIGO, 2021). Israel adopted this approach, and on January 28, 2021 advised pregnant women, especially those in the second and third trimesters, to be vaccinated (Ministry of Health, 2021). While studies around the world have been trying to assess the compliance of pregnant women, Israel already has actual statistics on vaccination rates, with 38.9% of its population having received two doses of the vaccine by March 3, 2021, and 52.7% by April 7, 2021 (Ministry of Health, 2021).

Irrespective of the issue of vaccination, high levels of stress and anxiety have been found among pregnant women during the pandemic (e.g., Lebel et al., 2020; Taubman – Ben‐Ari et al., 2020). This type of psychological distress is known to have adverse maternal and neonatal consequences, including low birth weight, premature deliveries, and unplanned operative deliveries (e.g., Grigoriadis et al., 2018). These perinatal outcomes also predict a number of risks for the child after birth, such as neonatal illness and mortality, developmental delays, and emotional and temperament difficulties, among others, accentuating the urgent importance of investigating the experience of pregnancy during the pandemic (Hu et al., 2021).

Pregnant women may be characterized by high levels of stress and anxiety during the crisis as a result of their twofold vulnerability as both pregnant and under the threat of COVID‐19 (Chasson et al., 2021). At the same time, however, they do not have definitive information as to the safety of receiving the vaccine, a fact that may intensify their anxiety even more. Consequently, we believed it was imperative to examine the possible contribution of vaccination status to pregnant women's anxiety levels and the contribution of the combination of vaccination status and various specific anxieties to their psychological distress. This study therefore compares psychological distress among pregnant Israeli women who had been infected by COVID‐19 and recovered, those who had received the vaccine, and those who had declined vaccination.

Israel is a multicultural society, with a Jewish majority and a large Arab minority (21% of the population). Compared to the Jewish population, Arab society tends to be more collective, with lower levels of education, income, and employment (Central Bureau of Statistics, 2019) and lower health conditions (Ministry of Health, 2018). Arab women in Israel have also been found to be more vulnerable and at greater risk of distress in the face of the spread of COVID‐19 (Kimhi et al., 2020; Taubman – Ben‐Ari et al., 2020). Moreover, a study regarding attitudes toward COVID‐19 vaccines in the general Israeli population indicated that more Arab than Jewish women reported that they would refuse the vaccine when it became available (Green et al., 2021). Distrustful attitudes toward vaccination were similarly reported to be higher among individuals from ethnic minority backgrounds in the United Kingdom (Paul et al., 2021). It is reasonable to assume that the attitudes found in the general public apply to pregnant women as well.

The current situation in Israel offers an opportunity to examine the effect of pregnant women's vaccination status in two subpopulations, Jewish and Arab. On the one hand, the two groups share the same medical services and are exposed to the same national campaign calling on everyone eligible, including pregnant women, to be vaccinated. On the other hand, however, they are characterized by different social norms regarding health issues.

We framed four research questions for this study: Are there ethnic differences in women's report of their vaccination status?; Are there differences in COVID‐19‐related anxieties among Arab women by vaccination status? What are the associations between the sociodemographic variables and COVID‐19‐related anxieties on the one hand and women's psychological distress on the other? What is the unique and combined contribution of the independent variables to the psychological distress of Arab women? Because of the novelty of the research questions and the explorative nature of the study, no hypotheses could be phrased.

## 3 METHODS

### 3.1 Participants and procedure

Following approval from the university's institutional review board (no. 032004/2), a sample of 860 Israeli women, 187 Jewish and 673 Arab, was recruited from March 3 to April 7, 2021. Recruitment of participants began about 1 month after a direct appeal by the Health Ministry to pregnant women to get vaccinated. A request to participate in the study was posted on social media groups for women in general and specifically for pregnant women, and links to electronic versions of the questionnaire in Hebrew and Arabic were provided. The opening page ensured the anonymity and confidentiality of the information and explained that the woman could cease to participate at any stage should she wish to do so. In addition, the women were informed that if they felt any distress during or after completing the questionnaire, they could call or email the researchers, whose contact details were supplied. Participants were considered eligible for the study if they were pregnant and indicated that they could complete questionnaires in Hebrew or Arabic. Of the 1328 women who started to respond to the questionnaire, 860 completed it in full (65% response rate). This response rate was similar for Jews and Arabs. It is worthy of note that many more Arab women chose to participate in the study, perhaps owing to the fact that whereas numerous studies are directed toward the Jewish population in Israel, less attention is paid to the Arab community, and thus they were less inundated by requests to participate in other studies. Nevertheless, power analysis, using G*power 3.1 (Faul et al., 2009), indicated that both subsample sizes exceeded the necessary minimum. To ensure that the power for a medium size effect (0.5) will be 0.80, a minimum sample size of 156 participants was required. However, we strove to recruit a larger number of participants in order to strengthen the power of the findings.

### 3.2 Instruments

A four‐part self‐administered questionnaire was developed for the study. Some sections were constructed specifically for this study, and others were drawn from previous studies conducted at early phases of the pandemic.

The first part of the questionnaire consisted of a sociodemographic inventory tapping the woman's background characteristics: age (continuous), education (1 = elementary; 2 = high school; 3 = post high school; 4 = academic), economic status (1 = below average; 2 = average; 3 = above average), physical health (1 = poor; 2 = average; 3 = good; 4 = very good), marital status (1 = single; 2 = married; 3 = in a couple relationship without marriage), ethnicity (0 = Jewish; 1 = Arab); gestation week (continuous), parity (0 = primiparous; 1 = multiparous), at‐risk pregnancy (0 = having been diagnosed with a particular risk factor for pregnancy; 1 = regular/not at‐risk), and whether or not the women had undergone fertility treatments to conceive (0 = spontaneous pregnancy; 1 = pregnancy following fertility treatments).

The second part contained five items tapping the participant's COVID‐19‐related vaccination status and her intentions in this regard. The women were asked if (1) they have been infected by COVID‐19 (yes, no), (2) they have received the vaccination (yes, no), (3) if yes to item 2, before or during pregnancy?, (4) if no to item 2, do they intend to be vaccinated? (yes, no), and (5) if yes to item 4, during pregnancy or after childbirth?

The third part contained nine items tapping the participants' COVID‐19‐related anxiety, as reflected in their perception of several aspects of the situation (Taubman – Ben‐Ari et al., 2020). The women were asked how much anxiety they felt about (1) the economic damage that may affect them and their family, (2) being infected by COVID‐19, (3) a family member being infected by COVID‐19, (4) being in public places, (5) using public transportation, (6) visiting hospitals or community clinics for pregnancy checkups, (7) the health of their fetus, (8) the delivery, and (9) raising the infant. Responses were marked on a scale from 1 (*very little*) to 5 (*very much*). Scores were given for each of the items separately, with higher scores indicating greater anxiety of that type.

The fourth part of the questionnaire consisted of the Mental Health Inventory—Short Form (MHI‐5; Stewart et al., 1988) based on the original MHI (Veit & Ware, 1983), which was used to assess psychological distress. The inventory consists of five items relating to the participant's well‐being (e.g., “I felt relaxed and stress free”) and distress (e.g., “I felt sad and upset”) during the past week. Responses were indicated on a 6‐point Likert scale ranging from 1 (*never*) to 6 (*all the time*). This short version of the inventory is widely used and has been found to have good internal consistency (Cronbach's alpha = 0.80–0.97; Kelly et al., 2008). In this study, Cronbach's alpha was 0.82, and thus a psychological distress score was calculated by averaging the responses to all five items (after adjusting for reverse‐coding), with a higher score reflecting greater psychological distress.

### 3.3 Data analysis

Data analysis was performed in three stages. First, descriptive statistics of the background characteristics were calculated for the sample as a whole, and both independent samples *t*‐tests and chi‐square tests were conducted to examine differences in background variables between the ethnic groups. Next, a series of independent samples *t*‐tests were computed to examine ethnic differences in women's report of their vaccination status. Finally, as most Jewish women had either recovered from the virus or been vaccinated, differences in COVID‐19‐related anxieties by vaccination status were calculated for the Arab sample alone, and Pearson correlations were computed between the sociodemographic variables and COVID‐19‐related anxieties on the one hand and women's psychological distress on the other. A three‐step hierarchical regression was then performed to determine the contribution of the independent variables to the psychological distress of Arab women. The variables were entered as follows: In Step 1, background variables; in Step 2, vaccination status (vaccinated vs. not vaccinated; recovered vs. not vaccinated); and in Step 3, the nine COVID‐19‐related anxieties. All analyses were conducted using SPSS (ver. 24).

## 4 RESULTS

### 4.1 Sociodemographic characteristics of the whole sample

The 860 women were aged 19–46 (M = 28.3, SD = 4.39) and in gestational weeks 4–42 (M = 24.66, SD = 9.81). Of these, 28.3% were expecting their first child, and 71.7% already had at least one child. Most of the participants were married or in a spousal relationship (97%); 62.9% had an academic degree, and the rest had a high school or post‐high school diploma; 81.2% defined their income as average, 13.4% as above average, and 5.5% as below average; and 47.6% defined their health status as very good, 39.8% as good, and the rest as average or poor. Ten percent of the participants reported that they had conceived through fertility treatments, and 23.1% reported that their pregnancy was considered at risk.

Sociodemographic characteristics by ethnicity appear in Table 1. As the table shows, among the Arab women, mean age, physical health, and proportion of academic education were significantly lower, and they had a lower rate of fertility treatments, than among the Jewish women.

**TABLE 1:** Sociodemographic characteristics by ethnicity

Variable	Jewish women (*n* = 187)	Arab women (*n* = 673)	Difference test *t*/χ^2^	*p* value
Age			**t (856) = 9.06**	**<0.001**
M	30.76	27.61		
SD	4.95	3.97		
Education %			**χ2 (3, 860) = 36.** **04**	**<0.001**
Elementary	0.0	0.0		
High school	7.0	19.3		
Post‐high school	12.3	22.6		
Academic	80.2	58.1		
Economic status %			**χ2 (2, 860) = 5.8**	0.055
Below average	8.6	4.6		
Average	75.9	82.6		
Above average	15.5	12.8		
Physical health %			**χ2 (3, 860) = 22.9**	**<0.001**
Poor	0.5	4.1		
Average	2.7	11.1		
Good	38.5	40.1		
Very good	58.3	44.6		
Fertility treatments %			**χ2 (1, 860) = 13.43**	**<0.001**
No	82.9	92		
Yes	17.1	8		
At‐risk pregnancy %			χ2 (1, 860) = 0.003	92.0
No	77	76.8		
Yes	23	23.2		
Parity %			χ2 (1, 860) = 3.48	062.0
Primiparous	33.7	26.7		
Multiparous	66.3	73.3		
Gestation week				
M	26.00	24.24	t (858) = 0.21	0.830
SD	9.28	10.07		

*Note:* The bold values are represents statistically significant.

### 4.2 Vaccination status by ethnicity

Among the pregnant women in the sample, 6.5% (*n* = 12) of the Jewish participants, and 23.6% (*n* = 159) of the Arab participants had recovered from the virus. Of those who had not already been infected, 93.6% of the Jewish women and 65.6% of the Arab women had received the vaccine. In the Jewish sample, average gestation week was 25.91 (SD = 7.99) for vaccinated women (*n* = 131), and 24 (SD = 11.58) for those who were not vaccinated (*n* = 9), t(138) = 0.67, *p* = n.s. In the Arab sample, average gestation week for vaccinated women was higher (*n* = 258; M = 26.54; SD = 9.86) than for those who were not vaccinated (*n* = 135; M = 23.08; SD = 10.68), t(391) = 3.21, *p* < 0.001. Among the vaccinated participants, 99.2% of Jewish women and 91% of the Arab women had received the vaccine during pregnancy. Among those who had not been vaccinated, 85.7% of the Jewish women reported the intention to receive the vaccine in the future (*n* = 6; 3 during pregnancy, and 3 after childbirth), and 70.5% of Arab women indicated the same intention (*n* = 91; 33, i.e., 37.1%, during pregnancy, and 56, i.e., 62.9%, after childbirth).

### 4.3 COVID‐19‐ related anxieties and psychological distress among Arab pregnant women by vaccination status

The mean level of psychological distress among the Jewish women in the sample was lower than that of the Arab women (M = 2.73, SD = 0.71; M = 3.29, SD = 0.87, respectively), t(854) = 7.904, *p* = 0.000. However, as most of the Jewish women were vaccinated (70.7%), and only a few had either already been infected by COVID‐19 (6.5%) or belonged to neither category (12.8%), we examined COVID‐19‐related anxieties and the contributors to psychological distress in the Arab sample alone. Here, 23.6% of the women had been infected and recovered, 34.8% were vaccinated, and 41.6% had neither recovered nor been vaccinated.

Table 2 presents the means and SDs of Arab participants' replies to the nine COVID‐19‐related anxiety items and the MHI. As Table 2 indicates, a number of differences were found between the groups: anxiety over being infected by the virus was higher among women who had recovered than in both other groups; anxiety over other family members being infected was higher in the vaccinated group than among the women who had recovered; anxiety over being in public places was higher among women who neither recovered nor been vaccinated than in the group of those who had recovered; and anxiety over using public transportation was higher among the vaccinated women than among women who had recovered. In addition, psychological distress was lower among vaccinated women than in the other two groups. No other significant differences were found between the vaccination status groups.

**TABLE 2:** COVID‐19‐related anxieties and psychological distress of Arab pregnant women by vaccination status

	Recovered (n = 159)		Vaccinated (n = 234)		Neither (n = 280)		
Anxiety over:	M	SD	M	SD	M	SD	F
Economic damage	3.22	1.00	3.13	1.01	3.17	1.05	0.47
Being infected by COVID‐19	3.60^a^	1.17	3.96^bc^	1.06	4.01^bc^	1.02	7.91***
Family member being infected by COVID‐19	3.93^a^	1.11	4.24^b^	0.89	4.10	0.99	4.49*
Being in public places	3.63^a^	1.05	3.85	1.04	3.96^c^	0.98	5.24**
Using public transportation	4.08^a^	1.14	4.41^b^	0.92	4.32	1.00	5.25**
Going for pregnancy checkups	3.31	1.09	3.44	1.12	3.54	1.07	2.28
Health of fetus	4.47	0.91	4.61	0.75	4.50	0.86	1.48
Delivery	3.38	1.13	3.53	1.17	3.64	1.07	2.58
Raising the baby after birth	3.41	1.14	3.32	1.24	3.33	1.13	0.30
Psychological distress	3.37^ac^	0.85	3.13^b^	0.87	3.37^ac^	0.83	6.13**

*Note*: *p < 0.05, **p < 0.01, ***p < 0.001.

### 4.4 The contribution of socioeconomic characteristics, vaccination status, and COVID‐19‐related anxieties to psychological distress

Pearson correlations between the sociodemographic characteristics and COVID‐19‐related anxieties on the one hand and psychological distress on the other appear in Table 3. As the table shows, lower physical health, lower economic status, being multiparous, and not being vaccinated all yielded significant, albeit relatively weak, associations with higher psychological distress. Furthermore, higher levels of all categories of COVID‐19‐related anxieties were significantly associated with higher psychological distress.

**TABLE 3:** Pearson correlations and hierarchical regression coefficients (Beta weights) for psychological distress among Arab pregnant women

	*Psychological distress*			
	*r*	*ß*	*t*	∆*R* ^2^
Step 1				0.066***
Age	0.32	0.02	0.46	
Education	−0.07	−0.023	−0.59	
Economic status	−0.12**	−0.08	−2.02*	
Physical health	−0.22***	−0.20	−5.15***	
Parity^a^	0.11**	0.09	2.24*	
Gestation week	−0.01	0.002	0.06	
Fertility treatments^b^	−0.01	−0.01	−0.29	
At‐risk pregnancy^c^	0.05	0.01	0.12	
Step 2				0.011***
Recovered vs. neither ^d^	0.05	0.02	0.46	
Vaccinated vs. neither ^e^	−0.13***	−0.10	−2.35*	
Step 3				0.126***
Economic damage	0.29***	0.180	4.48***	
Being infected by COVID‐19	0.17***	0.0040	0.07	
Family member being infected by COVID‐19	0.16***	0.25	9.49***	
Being in public places	0.15***	0.04‐	−0.80	
Using public transportation	0.12***	0.020‐	−0.45	
Going for pregnancy checkups	0.21***	0.07	1.40	
Health of fetus	0.13***	0.03‐	0.56	
Delivery	0.28***	0.12	2.38*	
Raising the baby after birth	0.28***	0.13	2.75**	
R^2^				20.3
*F* (19, 670)				8.73***

*Note*: ^a^0 = primiparous, 1 = multiparous; ^b^ 0 = No, 1 = Yes; ^c^ 0 = No, 1 = Yes; ^d^ 0 = Neither, 1 = Recovered; ^e^ 0 = Neither, 1 = Vaccinated.

*Note*: *p < 0.05, **p < 0.01, ***p < 0.001.

The regression analysis for psychological distress revealed that the independent variables explained 20.3% of the variance in this outcome. The results are presented in Table 3.

As can be seen from Table 3, background characteristics in Step 1 contributed a significant 6.6% to the explained variance, with poorer health, lower economic status, and already being a mother contributing significantly to higher psychological distress. Vaccination status in Step 2 added 1.1% to the explanation of the variance, with those who were not vaccinated reporting higher psychological distress. Step 3 contributed an additional 12.6% to the explained variance, with higher anxieties over economic damage, a family member being infected by the virus, delivery, and raising the baby significantly associated with higher psychological distress.

## 5 DISCUSSION

Israel's early and rapid vaccination program offers a unique opportunity to examine pivotal questions relating to issues of compelling importance in the current pandemic, among them, pregnant women's anxieties and psychological distress in view of their vaccination status. As this study shows, fewer Jewish women were infected by the virus than Arab women, and a higher proportion was vaccinated. This is in line with previous studies indicating that ethnic minorities in general, and Arab women in Israel in particular, are less willing to receive the vaccine (Green et al., 2021; Paul et al., 2021), which may reflect their higher distrust of the government (Paul et al., 2021). As the majority of Jewish women in our sample were either vaccinated or had been infected and recovered, we concentrated here on the factors associated with psychological distress among Arab women. Moreover, in line with previous studies (Kimhi et al., 2020; Taubman – Ben‐Ari et al., 2020), psychological distress was higher among the Arab women than among their Jewish counterparts.

Our main finding is the lower level of psychological distress among Arab women who had been vaccinated compared to those who had recovered or had chosen not to receive the vaccine. This might indicate a sense of security as a result of conforming to medical advice, which recommended that pregnant women be vaccinated in order to protect the pregnancy and fetus. Although we cannot draw any causal conclusions from the results, they may provide initial evidence that vaccination relieves some of the burden of worries in this vulnerable period when the health of the pregnant woman and her fetus is threatened. This suggestion may be strengthened by the lower psychological distress found among the Jewish women, most of whom were vaccinated.

Interestingly, whereas vaccinated women were less anxious about being in public places than those who had neither recovered nor been vaccinated, they were more anxious about using public transportation than those who had been infected and recovered. This may be a logical reaction to the reality that existed at the time of this study. On the one hand, more and more public places were open to the general public, some only to those with what was known as a “green pass,” that is, people who had recovered or been vaccinated. On the other hand, however, public transportation was still limited, and the services that operated, such as buses and taxies, did so without any restrictions (i.e., without the green pass). It is reasonable to assume that women who were more aware of the threat of COVID‐19 were particularly concerned about being in close contact with people who might be capable of passing on the infection.

Another interesting finding is that women who were vaccinated were more anxious about being infected than the other two groups, and more anxious about the possible infection of other family members than those who had recovered. This appears to be counterintuitive. However, it might indicate a degree of mistrust in the vaccine in this population, so that those who had already recovered from COVID‐19 felt more immune to the virus. Another possibility is that anxiety about COVID‐19 was a motivating factor to obtain vaccination against COVID‐19, as has been suggested in a systematic review concerning the association between COVID‐19 anxiety of the extent of compliance to be vaccinated against it among pregnant women (Januszek et al., 2021). As this is the first study to explore this issue, future studies are warranted to deepen our understanding of the psychological mechanisms underlying pregnant women's fears of infection.

Furthermore, lower economic status and physical health and already being a mother were related to higher psychological distress among the Arab women. The findings regarding economic status and physical health are consistent with the results of previous studies (Gur et al., 2020; Januszek et al., 2021; Matsushima & Horiguchi, 2020) and may indicate that less favorable background characteristics, which put pregnant women at greater risk for psychological distress even in routine times (Goletzke et al., 2017; Koleva et al., 2011), are even more telling after prolonged coping with a pandemic that has serious implications for their health, social, and economic status. In the case of mothers, the need to care for their children while contending with the pandemic and their pregnancy concurrently may place enormous demands on them and exhaust their ability to cope well with the situation. This is in line with a previous study (Effati‐Daryani et al., 2020), in which the authors recommend increasing mothers' access to health centers, which may provide support and important information. Other studies, however, have found that women pregnant with their first child were more anxious during the pandemic than those who were already mothers (e.g., Lebel et al., 2020; Mortazavi et al., 2021).

Although all the examined COVID‐19‐related anxieties were associated with higher psychological distress, examination of their concurrent contribution to this outcome beyond the sociodemographic variables and vaccination status revealed that only concerns regarding the delivery and raising the baby after birth made a significant contribution. The women seem to have been less preoccupied by present reality and more anxious about the future. This is in line with a study conducted among pregnant women in the United States (Lebel et al., 2020), in which higher levels of depression and anxiety were associated with greater concerns about the effect of COVID‐19 on prenatal care and the life of the mother and baby after birth.

This study is among the first to investigate the associations between the vaccination status of pregnant women and psychological distress and several COVID‐19‐related anxieties. However, a number of limitations should be noted. First, the study relies on a convenience sample that cannot be considered representative of the population of pregnant women in Israel. Second, in terms of vaccination status, the Jewish sample was quite homogenous, as a large proportion of the women had received the vaccine. This situation, which was undoubtedly a result of the highly dynamic and unpredictable nature of the pandemic and the measures taken to prevent it, made it impossible for us to examine the link between vaccination status and psychological distress in this subsample. Nevertheless, we believe that our impressive sample of Arab women, in which the three vaccination groups were represented, was a unique and valuable asset that assisted in shedding light on the relationship between vaccination status, COVID‐19‐related anxieties, and psychological distress among pregnant women in general and in an ethnic minority in particular.

## 6 CONCLUSIONS

Despite these limitations, this study is very timely and is, to the best of our knowledge, the first to take advantage of the nationwide vaccination program in Israel to examine the distress and anxiety of pregnant women by vaccination status. The results showing that vaccinated women report the lowest level of psychological distress may be used as a tool for nurses in their efforts to convince women to get vaccinated, as well as in public campaigns to promote vaccination in this population. The difference found in the extent of vaccination between Jewish and Arab women highlights the need for a more culturally sensitive approach in the attempt to encourage vaccination in diverse ethnic groups, that is, a campaign that takes into account different needs, concerns, and attitudes. Further in‐depth investigation of the issue addressed here may help countries around the world in their efforts to curb the pandemic.

## CONFLICT OF INTEREST

The authors have no conflict of interest to declare.

## AUTHOR CONTRIBUTIONS

*Study design*: Orit Taubman – Ben‐Ari, Efrat Weiss, Salam Abu Sharkia. *Data collection*: Efrat Weiss, Salam Abu Sharkia, Enas Khalaf. *Data analysis*: Orit Taubman – Ben‐Ari. *Manuscript writing*: Orit Taubman – Ben‐Ari.

## ETHICS STATEMENT

The study was conducted at Bar‐Ilan University. Ethical approval was granted by the School of Social Work at Bar‐Ilan university's institutional review board.

## Data Availability

The data that support the findings of this study are available from the corresponding author upon reasonable request.
